# Survival of Patients with Metastatic Melanoma Treated with Ipilimumab after PD-1 Inhibitors: A Single-Center Real-World Study

**DOI:** 10.3390/cancers16193397

**Published:** 2024-10-04

**Authors:** Sofia Verkhovskaia, Rosa Falcone, Francesca Romana Di Pietro, Maria Luigia Carbone, Tonia Samela, Marie Perez, Giulia Poti, Maria Francesca Morelli, Albina Rita Zappalà, Zorika Christiana Di Rocco, Roberto Morese, Gabriele Piesco, Paolo Chesi, Paolo Marchetti, Damiano Abeni, Cristina Maria Failla, Federica De Galitiis

**Affiliations:** 1 Department of Oncology, Istituto Dermopatico dell’Immacolata IDI-IRCCS, 00167 Rome, Italy; s.verkhovskaia@idi.it (S.V.); r.falcone@idi.it (R.F.); f.dipietro@idi.it (F.R.D.P.); g.poti@idi.it (G.P.); m.morelli@idi.it (M.F.M.); a.zappala@idi.it (A.R.Z.); z.dirocco@idi.it (Z.C.D.R.); r.morese@idi.it (R.M.); g.piesco@idi.it (G.P.); p.chesi@idi.it (P.C.); p.marchetti@idi.it (P.M.); f.degalitiis@idi.it (F.D.G.); 2 Clinical Trial Center, Istituto Dermopatico dell’Immacolata IDI-IRCCS, 00167 Rome, Italy; 3 Epidemiology Units, Istituto Dermopatico dell’Immacolata IDI-IRCCS, 00167 Rome, Italy; t.samela@idi.it (T.S.); d.abeni@idi.it (D.A.); 4 Department of Histopathology, Istituto Dermopatico dell’Immacolata IDI-IRCCS, 00167 Rome, Italy; m.perez@idi.it; 5 Experimental Immunology Laboratory, Istituto Dermopatico dell’Immacolata IDI-IRCCS, 00167 Rome, Italy; c.failla@idi.it

**Keywords:** melanoma, immunotherapy, BRAF, NRAS, ipilimumab

## Abstract

**Simple Summary:**

This research was conducted to evaluate the impact of ipilimumab treatment in patients with metastatic melanoma when monotherapy with PD-1 inhibitors has failed. In particular, the aim was to evaluate the efficacy of ipilimumab in this setting based on the presence or absence of BRAF or NRAS mutations. The present study could allow us to understand when salvage treatment with ipilimumab would have the best impact, although an analysis on a larger patient cohort would be necessary.

**Abstract:**

Background: When monotherapy with PD-1 inhibitors in metastatic melanoma fails, there are currently no standard second-line choices. In case of the unavailability of clinical trials, ipilimumab represents a possible alternative treatment. Methods: We collected data of 44 patients who received ipilimumab after the failure of PD-1 inhibitors from July 2017 to May 2023 at our Institute. Overall survival (OS), progression-free survival (PFS), and post-progression survival (PPS) based on BRAF or NRAS mutation status, sex, and the presence of brain metastases were estimated using the Kaplan–Meier method. Cox regression was used to evaluate independence in multivariate analysis. The objective response rate (ORR) was estimated based on RECIST 1.1. Results: Among the 44 patients enrolled in this study, 28 BRAF-wildtype, 9 BRAF-mutated, and 7 NRAS-mutated patients were identified. OS analysis showed a significant difference between wildtype and BRAF- or NRAS-mutated patients: 23.2 months vs 5.3 and 4.59, respectively, *p* = 0.017. The presence of brain metastases and BRAF or NRAS mutation were independent factors for mortality in multivariate analysis. Conclusions: In case of failure to enroll patients in innovative clinical trials, second-line ipilimumab still represents an effective therapy in patients with metastatic wildtype melanoma and in the absence of brain metastases.

## 1. Introduction

The immunotherapy new era, characterized by the usage of the cytotoxic T lymphocyte-associated protein (CTLA)-4 inhibitor ipilimumab and of programmed cell death receptor (PD)-1 inhibitors (nivolumab and pembrolizumab), brought important changes in the treatment of patients with metastatic melanoma, improving overall responses and achieving a 5-year survival rate of approximately 40% in treated patients [1,2]. The combination of ipilimumab and PD-1 inhibitors (iPD-1) is even more effective, with significative positive responses and an overall survival rate of 52% at 5 years [3]. However, due to regulatory restrictions that have limited combination treatment to patients with brain metastases and/or with expression of PD-1 ligand (PD-L1) < 1%, many patients in Italy cannot benefit from this combined therapy. Moreover, this combination treatment has only been available in Italy since 2022.

When combined treatment is not possible or after the failure of BRAF and MEK inhibitors in BRAF-mutated melanomas, approximately 75% of patients treated with monotherapy with iPD-1 undergo progression within 5 years [2]. BRAF-mutated patients may also benefit from treatment with BRAF and MEK inhibitors after iPD-1 therapy, but for BRAF-wildtype patients who progress during iPD-1 therapy and patients with BRAF mutation who fail both target therapy and treatment with iPD-1, there is a lack of additional effective treatments. The enrollment of these patients in clinical trials with innovative therapies represents the best clinical choice, but in case of the unavailability of such studies or an inability to enroll patients who do not satisfy the inclusion criteria, treatment with the CTLA-4 inhibitor ipilimumab represents a possible option.

Ipilimumab was the first immunotherapeutic drug approved for the treatment of metastatic melanoma in 2011, radically changing the landscape of this disease [4]. However, only a subset of patients benefited from this treatment, both among pre-treated and untreated patients [5,6]. Several studies have shown the efficacy of treatment with ipilimumab in case of failure of previous iPD-1 therapy [7,8]. GV Long et al. evaluated patients from the KEYNOTE-006 study who had received treatment with ipilimumab in monotherapy after failure of pembrolizumab. Their study showed antitumor activity of ipilimumab with a median overall survival (OS) from randomization of 19.6 months [7]. L Zimmer et al. evaluated patients treated with ipilimumab monotherapy or a combination of ipilimumab and nivolumab after failure of therapy with iPD1. In the ipilimumab monotherapy arm, the one-year survival rate was 54% and the objective response rate (ORR) was 16% [8].

Recent research has highlighted the potential advantages of combining ipilimumab with iPD-1 in treating patients who have shown disease progression after iPD-1 monotherapy. This combination therapy has been shown to enhance the immune response against tumors more effectively than ipilimumab alone. However, despite these promising results, the combination therapy is not yet widely available outside of clinical trial settings. Ongoing studies aim to further evaluate the safety, efficacy, and potential long-term benefits of this treatment approach, with the hope of making it accessible to a broader patient population in the future [9,10,11].

NRAS mutation in melanoma seems to be a prognostic marker of worse prognosis and more aggressive disease, with several studies indicating that OS for patients with NRAS-mutant melanoma is significantly lower compared to those with BRAF-mutant or NRAS/BRAF-wildtype tumor status [12,13]. NRAS mutation is quite frequent in melanoma; it is present in about 15–20% of patients with advanced disease [14] and is probably responsible for the upregulation of the mitogen-activated protein kinase (MAPK) pathway, which can promote cell proliferation and tumor cell survival, leading to increased tumor aggressiveness [15,16]. Its role as a predictor of the efficacy of immunotherapy is uncertain, and data in the literature are conflicting. In a study by Zhou L [17], the presence of NRAS mutation was associated with a poorer response and worse prognosis in patients with advanced melanoma treated with iPD-1. Another study showed comparable ORR but lower OS in NRAS-mutated advanced melanomas treated with ipilimumab, iPD-1 alone, or iPD-1 and ipilimumab in combination [18]. In an Italian study on NRAS-mutated patients with advanced melanoma treated with first-line immunotherapy, no significant differences were observed in ORR, disease control rate (DCR), progression-free survival (PFS), or OS [19]. Conversely, a recent meta-analysis showed a positive impact of NRAS mutation on immunotherapy responses, with a greater probability of achieving an objective response in these patients, but impact on OS was not assessed in that study [20].

Based on these data, we decided to carry out a retrospective study evaluating the predictive role of NRAS and BRAF mutations in patients at our Institute treated with ipilimumab in any line of treatment after the failure of iPD-1 therapy. Our aim was to estimate the impact of NRAS and BRAF mutations on the OS, PFS, post-progression survival (PPS), and ORR of these patients.

## 2. Materials and Methods

### 2.1. Patients

In this study, we included patients aged ≥ 18 years who had inoperable or metastatic melanoma according to the American Joint Committee on Cancer melanoma staging and classification (AJCC 8) [21] and who underwent therapy with ipilimumab in any line of treatment after failure of iPD1. Patients who received ipilimumab after adjuvant iPD-1 treatment failure were also included. All patients had unsuccessful treatment with iPD-1 regardless of the previous treatments carried out. BRAF-mutated patients who had prior treatment with BRAF and MEK inhibitors were also included. Data were available for 44 consecutive patients followed at our Center from July 2017 to May 2023. The study protocol was approved by the IDI-IRCCS Institutional Ethical Committee (n. 510/3, 2018). Information on age, sex, comorbidities, disease location and stage, site of primary melanoma, lactate dehydrogenase (LDH) level, prior adjuvant, or metastatic treatment, ipilimumab treatment line, number of cycles performed, mutation status, and reported toxicities was noted from medical records.

The primary endpoint was OS, defined as the time from initiation of ipilimumab treatment to death, loss to, or end of follow-up. The secondary endpoints were PFS, defined as the time from initiation of ipilimumab treatment to progression, PPS, defined as the time from the date of progression to death, loss to, or end of follow-up [22], and ORR, defined as the best response to treatment.

**Figure 1:**
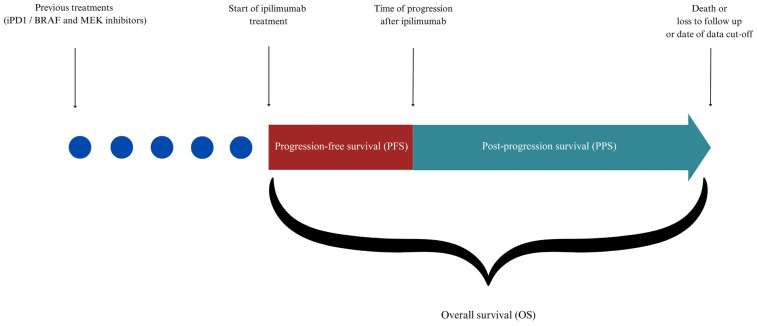
Time scheme used for calculation of overall survival (OS), progression-free survival (PFS), and post-progression survival (PPS).

Progression data were collected from multiple sources, including physical exams at unscheduled visits and radiological evaluations. ORR was assessed using the Response Evaluation Criteria in Solid Tumors (RECIST) version 1.1 [23]. The OS, PFS, and PPS calculation scheme is reported in Figure 1. Evaluation of NRAS and BRAF mutations was performed by multiplex allele-specific real-time PCR using the commercial kit “BRAF/NRAS mutation test” (Roche Diagnostics, Rotkreuz, Switzerland). The test is designed to detect clinically relevant mutations in the NRAS (exons 2, 3, and 4) and BRAF (exons 11 and 15) genes. Specific mutations were successively confirmed by the Sanger sequencing method.

### 2.2. Statistical Analysis

Descriptive statistics with frequencies, mean values, and standard deviation, or median values and interquartile ranges (IQRs), were used to describe demographic and clinical patient characteristics. The Kaplan–Meier product limit estimator was used to estimate the survival functions and the log–rank test was used to compare the survival curves among different clinical groups. The Cox proportional hazard model was used to estimate the association between BRAF and NRAS mutation status and risk of mortality while controlling for potential confounders, such as sex and presence of brain metastases. Hazard Ratios (HRs) and their 95% confidence intervals (CIs) were calculated.

## 3. Results

A total of 44 consecutive patients were included in this study. As shown in Table 1, most patients were males (*n* = 27, 62%); there were 17 females (38%). The median age at baseline was 68 years (range, 36–90 years old).

The majority of primary tumors were of cutaneous origin, accounting for a total of 35 patients (80%). Four patients had mucosal melanoma (9%), one patient (2%) had uveal melanoma, and one patient (2%) had acral melanoma. For the remaining three patients (7%), the origin was unknown (melanoma of unknown primary).

Among the 44 patients, 28 BRAF-wildtype, 9 BRAF-mutated, and 7 NRAS-mutated patients were observed (64%, 20%, and 16% of the study population, respectively).

NRAS mutation data were not available for three BRAF-wildtype patients. Of the BRAF-mutated patients, seven had a BRAFV600E mutation, and one had a BRAFV600K mutation; for one patient, data on sequencing were not known.

Regarding metastatic sites before treatment initiation with ipilimumab, 21 patients (48%) had one or two sites of metastasis, while 23 patients (52%) had more than two sites of metastasis.

Only four patients (9%) had brain metastases at baseline before ipilimumab treatment and two of them had received stereotactic radiotherapy treatment for metastatic lesions. All BRAF-mutated patients received a prior line of BRAF and MEK inhibitors before or after iPD-1 treatment and five patients received chemotherapy before receiving ipilimumab treatment. “PFS to prior therapy with BRAF and MEK inhibitors was 11 months (CI: 2.2–19.7) in the nine mutated patients reflecting the data in the literature [24] (Supplementary Figure S1).

**Figure 2:**
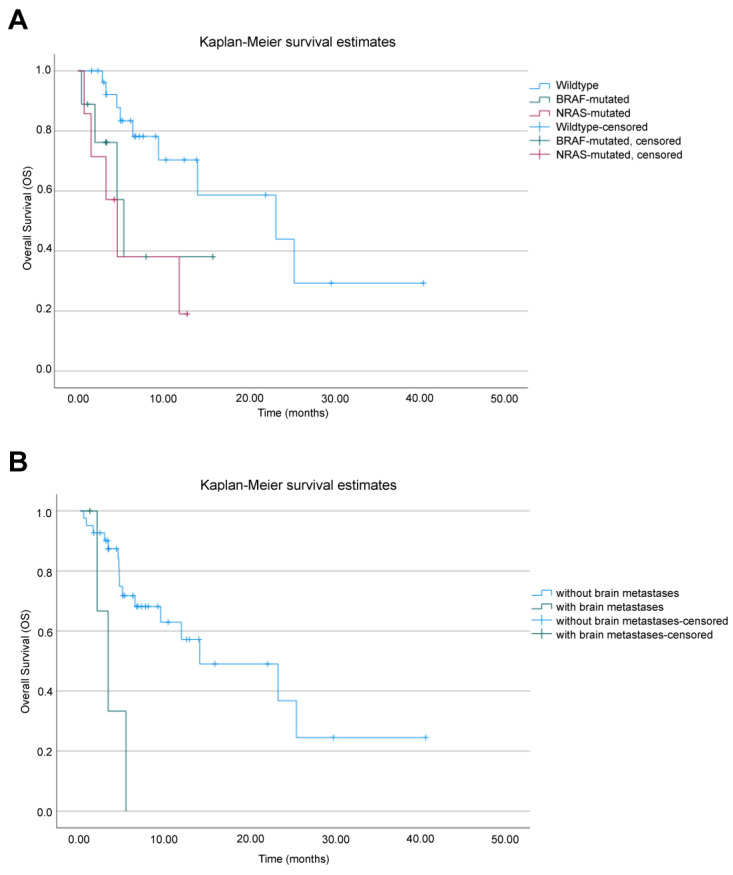
Overall survival (OS) analysis. Kaplan–Meier survival curves in patients who received ipilimumab treatment after anti-PD-1 therapy failure, based on (**A**) presence of BRAF or NRAS mutation and (**B**) presence of brain metastases.

In the study population, the median OS was 14 months (95% CI: 2.1–25.8), with 23.2 months for BRAF-wildtype (95% CI: 2.9–43.4), 5.3 months for BRAF-mutated (95% CI: 3.6–7), and 4.5 months for NRAS-mutated patients (95% CI: 1.8–7.3) (*p* = 0.017) (Figure 2A). Median OS was not reached for female patients and was 11.8 months (95% CI: 2.8–20.9) for male patients (*p* = 0.130). Regarding brain metastases, median OS was 14 months (95% CI: 1.6–26.3) for patients without and 3.2 months (95% CI: 1.2–5.3) for patients with brain metastases, *p* = 0.006 (Figure 2B).

Cox proportional hazards analysis showed a higher mortality risk for mutated patients (BRAF or NRAS) and for patients with brain metastases, with an HR of 3.3 (95% CI: 1.1–9.3; *p* = 0.027) for patients with BRAF or NRAS mutations compared to BRAF wildtype, with an HR of 4.5 (95% CI: 1.0–19.1; *p* = 0.037) for patients with brain metastases, and a non-statistically significant HR of 2.8 (95% CI: 0.8–9.0; *p* = 0.089) for male patients compared to females (Table 2). Therefore, BRAF or NRAS mutations were independent risk factors for OS, after controlling for sex and the presence of brain metastases. Age was not associated with the outcome of interest, and it did not act as a confounder.

We then evaluated long surviving subjects and found that three patients had a survival of more than two years after starting ipilimumab: one patient had disease in the skin/subcutaneous tissue and lymph nodes; the second patient had disease in the skin/subcutaneous tissue and soft tissues. The third patient had disease in the lungs, liver, skin/subcutaneous tissue, and bone at the moment of starting ipilimumab treatment.

**Figure 3:**
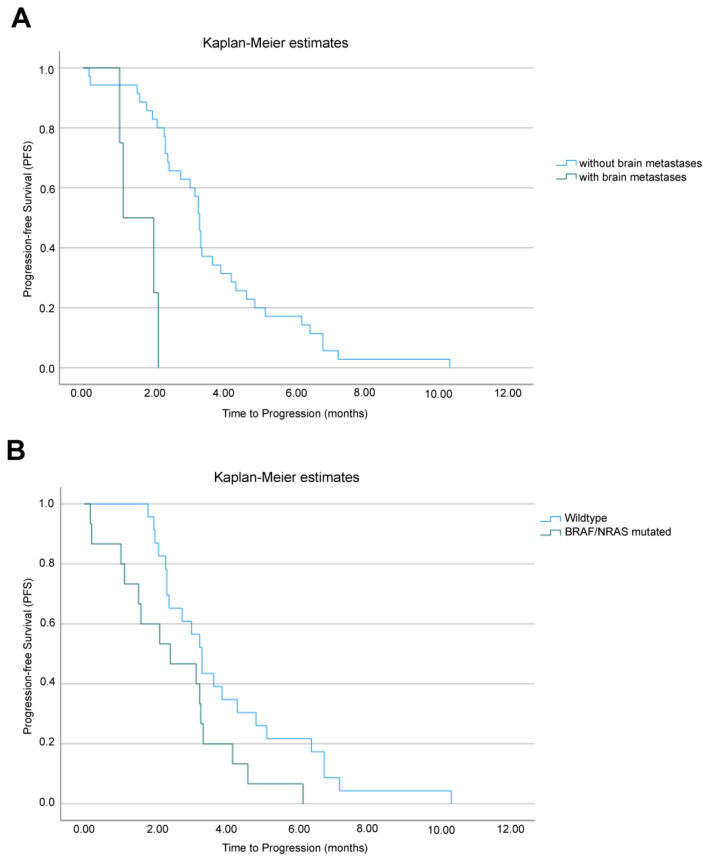
Progression-free survival (PFS) analysis. Kaplan–Meier curves in patients who received ipilimumab treatment after anti-PD-1 therapy failure based on (**A**) presence of brain metastases and (**B**) presence of pooled BRAF or NRAS mutation.

PFS was also evaluated, showing a median PFS of 3.2 months (95% CI: 2.7–3.7) for all patients. Median PFS was 1.1 months (95% CI: 1–2.0) for patients with brain metastases and 3.2 months (95% CI: 3.1–3.4) for patients without brain metastases, *p* < 0.001 (Figure 3A). No difference in PFS was seen regarding sex. A small difference in PFS was found between BRAF-wildtype patients and the pool of BRAF/NRAS-mutated patients. In fact, median PFS was 3.3 months (95% CI: 2.8–3.7) for BRAF-wildtype patients and 2.4 months (95% CI: 0.4–4.3) for pooled BRAF/NRAS-mutated patients, *p* = 0.033 (Figure 3B).

**Figure 4:**
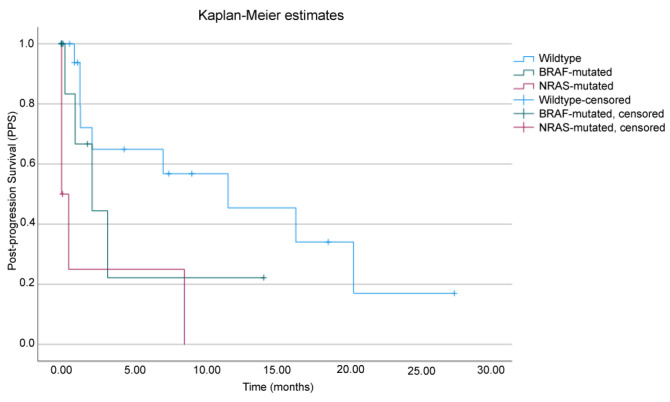
Post-progression survival (PPS) analysis. Kaplan–Meier survival curves in patients who received ipilimumab treatment after anti-PD-1 therapy failure based on the presence of BRAF or NRAS mutation.

Given the data discrepancy between OS and PFS, with little difference seen in PFS compared to a significant difference in OS, we evaluated PPS in order to estimate the true significance of the impact of PFS on OS. The median PPS was significantly higher in BRAF-wildtype in respect to BRAF- or NRAS-mutated patients, with 7.1 months (95% CI: 1.0–13.2) for all patients, 11.7 months (95% CI: 0–23.6) for wildtype patients, 2.1 months (95% CI: 0–4.4) for BRAF-mutated patients, and 0.0 months for NRAS-mutated patients, *p* = 0.002 (Figure 4).

Regarding ORR, in the BRAF-wildtype patient arm, eight patients (28.5%) had PR, two patients (7.1%) SD, and seventeen patients (60.9%) PD as best response. Conversely, of the nine BRAF-mutated patients, eight patients (88.9%) had PD as best response, and one patient (11.1%) had PR. Of the seven NRAS-mutated patients, the objective responses showed PD in five patients (71.4%), CR in one patient (14.3%), and PR in one patient (14.3%) (Table 3).

## 4. Discussion

We conducted a longitudinal non-concurrent real-world study on 44 patients with advanced (metastatic or unresectable) melanoma treated with ipilimumab after failure of iPD-1 treatment.

We found a significant difference in terms of OS in BRAF- and NRAS-mutated compared to BRAF-wildtype patients treated with ipilimumab after iPD1 treatment failure, with a higher OS in the wildtype group. In addition, a lower rate of progression was observed in wildtype patients compared to mutated patients.

Metastatic melanoma patients are currently treated with iPD-1 monotherapy or with BRAF and MEK inhibitors in the presence of activating BRAF mutations when combined iPD1 and iCTLA4 therapy is not possible. An optional therapy in the case of the failure of these treatments and of no possibility of enrollment in a clinical trial is presently represented by ipilimumab in monotherapy. In Italy, first-line treatment with the combination of ipilimumab and iPD-1, despite being more effective, with significant positive responses in 60% of patients [3], is in fact limited to patients with brain metastases and/or with expression of PD-L1 < 1%. Unfortunately, it is known from the literature that the effectiveness of ipilimumab in advanced lines of treatment is minimal [7].

As far as we know, there are no prospective studies that evaluated the efficacy of ipilimumab monotherapy in BRAF- and NRAS-mutated patients compared to wildtype individuals after failure of iPD1 treatment. Recently, several trials have been conducted to compare ipilimumab monotherapy versus rescue ipilimumab plus an iPD-1 after progression from first-line iPD-1 [8,9,10,11]. In the study of Pires da Silva et al., a subgroup analysis was performed based on BRAF or NRAS mutations to evaluate the efficacy of the ipilimumab plus iPD-1 combination versus ipilimumab alone, but in the ipilimumab-only arm, the difference in terms of efficacy based on the presence of BRAF or NRAS mutation was not evaluated [9].

In this study, we provide evidence that the presence of NRAS or BRAF V600 mutations serves as a negative prognostic indicator for patients undergoing ipilimumab monotherapy. Specifically, regarding BRAF V600 mutation, our findings pertain to patients who have previously been treated with BRAF and MEK inhibitors and have subsequently experienced disease progression following PD-1 inhibitor therapy. The data suggest that these genetic mutations are associated with poorer treatment outcomes, highlighting the need for alternative therapeutic strategies in this subset of patients.

Studies conducted to verify difference in predicting response to immunotherapy in patients with NRAS mutations are conflicting. Differently from the data obtained in our analysis, a recent meta-analysis demonstrated a positive predictive role for the ORR of this mutation in patients treated with immunotherapy, although no impact on OS was found [20]. This difference could be explained by the fact that our analysis was performed in the setting of subsequent lines of immunotherapy treatment.

In a retrospective study on 116 patients in which the efficacy of ipilimumab after treatment with iPD-1 was evaluated, subgroup analysis showed greater efficacy of ipilimumab in second-line immunotherapy in BRAF-mutated patients [25]. However, differently from our study, only 12 out of 116 patients were BRAF-mutated, and these 12 patients had not been pre-treated with BRAF and MEK inhibitors [25].

The small difference in PFS found in our population, in discordance with the large difference in OS, indicates that, once progression occurs in mutated patients, the disease is rapidly fatal, radically compressing the times of observation and consequently the possible differences between survival times. In any case, PPS analysis showed a significant difference in survival after progression in BRAF-wildtype patients (11.7 months) compared to BRAF (2.1 months)- or NRAS (0.0 months)-mutated ones. Therefore, patients harboring a BRAF or NRAS mutation seem to have shorter survival times compared to wildtype patients once that progression of disease occurs.

It may be possible that the positive impact of the NRAS mutation on treatment with iPD-1 may diminish once the disease progresses. This progression could unmask the mutation’s role as a negative prognostic factor, as documented in the literature. Initially, patients with NRAS mutations might respond well to iPD-1 therapy, but over time, the mutation’s adverse effects could become more apparent, leading to poorer outcomes during treatment with ipilimumab.

Further studies are necessary to better evaluate the impact of NRAS and BRAF mutations on the response to ipilimumab in melanoma and to define the best treatment for these patients after iPD1 therapy failure.

The impact on OS when brain metastases are present is well known in the literature.

The integration of immunotherapy with radiotherapy appears to be a safe and viable therapeutic approach and this combined modality demonstrates encouraging survival outcomes [26]. In our cohort, only two of the four patients with metastatic cerebral involvement received radiotherapy treatment. Greater integration of systemic treatments with locoregional therapies, such as radiotherapy, could improve the outcomes for these patients.

Even though non-statistically significant, we also found a trend in sex-based difference in treatment effectiveness in favor of females. The sex-based difference in survival favoring females in melanoma is well-documented in the literature [27], although studies on outcomes during immunotherapy are conflicting [28,29,30].

## 5. Conclusions

Despite the small sample size, our results indicate that a careful evaluation should be performed by clinicians when prescribing ipilimumab as treatment after the failure of therapy with iPD-1. The balance between the benefits and detrimental effects of ipilimumab therapy should be particularly defined in patients with activating BRAF mutations who have been pre-treated with BRAF and MEK inhibitors, as well as in NRAS-mutated patients. Special care should be adopted when a patient with brain metastases is to be treated. Instead, ipilimumab treatment could be of great value in patients with BRAF/NRAS-wildtype melanoma and without brain metastases, with several benefits to long-term survival, although a larger dataset is needed for a more precise indication.

## Figures and Tables

**Table 1:** Patient clinical characteristics.

	*n* = 44 pts ^a^	%
Age, y (range)	68	(36–90)
Sex		
Female	17	38
Male	27	62
Primary tumor		
Cutaneous	35	80
Mucosal	4	9
Uveal	1	2
Acral	1	2
Unknown	3	7
Mutations		
BRAF V600E	7	16
BRAF V600K	1	2
BRAF V600 ^b^	1	2
NRAS	7	16
NRAS unknown	3	7
No mutation	25	57
Previous treatment		
anti-PD-1	44	100
anti-BRAF/MEK inhibitors	9	20
Chemotherapy	5	11
Metastatic sites, *n*		
>2	23	52
1–2	21	48
Brain metastases		
Yes	4	9
No	40	91
Brain radiotherapy		
Yes	2	5
No	42	95

^a^ Median (IQR), ^b^ unknown BRAF sequencing.

**Table 2:** Prognostic factors for mortality: multivariate Cox model.

	All Patients (*n* = 44)	
	HR ^a^ (95% CI)	*p*
Sex		
Female	1	
Male	2.8 (0.9–9.1)	0.089
Brain metastases		
No brain metastases	1	
Brain metastases	4.6 (1.1–19.2)	0.037
Mutation status		
Wildtype	1	
NRAS or BRAF mutation	3.3 (1.1–9.3)	0.027

^a^: Hazard Ratio (HR) adjusted for all variables in the table.

**Table 3:** Prognostic factors for mortality: multivariate.

ORR (%)	CR	PR	SD	PD	NA
BRAF mut (*n* = 9)	0	11.1	0	88.9	0
NRAS mut (*n* = 7)	14.3	14.3	0	71.4	0
BRAF/NRAS WT (*n* = 28)	0	28.5	7.1	60.9	3.5

Abbreviations: ORR: overall response rate; CR: complete response; PR: partial response; SD: stable disease; PD: progressive disease; NA: not available; WT: wildtype.

## Data Availability

The data presented in this study are available on request from the corresponding author.

## Supplementary Materials

The following supporting information can be downloaded at https://www.mdpi.com/article/10.3390/cancers16193397/s1, Figure S1. Progression-free survival analysis.
