# Supplementary material for: Survival of Patients with Metastatic Melanoma Treated with Ipilimumab after PD-1 Inhibitors: A Single-Center Real-World Study
Source: Cancers (Basel). 2024 Oct 4;16(19):3397. doi: 10.3390/cancers16193397 (PMC11475497; doi:10.3390/cancers16193397)
Supplement: Supplementary file 1 [file cancers-16-03397-s001.zip › cancers-3169082-supplementary.pdf]

Supplementary Materials: Survival of Patients with Metastatic Melanoma Treated with Ipilimumab after PD-1 Inhibitors: A Single-Center Real-World Study

Sofia Verkhovskaia, Rosa Falcone, Francesca Romana Di Pietro, Maria Luigia Carbone, Tonia Samela, Marie Perez, Giulia Poti, Maria Francesca Morelli, Albina Rita Zappalà, Zorika Christiana Di Rocco, Roberto Morese, Gabriele Piesco, Paolo Chesi, Paolo Marchetti, Damiano Abeni, Cristina Maria Failla and Federica De Galitiis

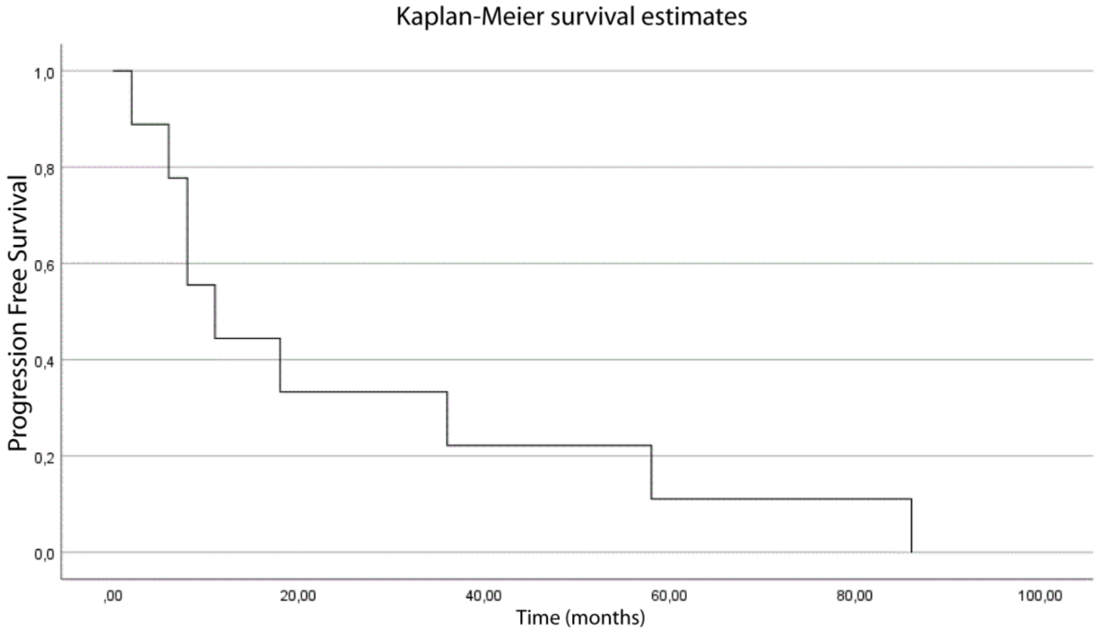

Figure S1. Progression Free Survival analysis. Kaplan-Meier survival curves in patients who performed BRAF and MEK inhibitors treatment.
